# “A Real Bucket of Worms”: Views of People Living with Dementia and Family Members on Supported Decision-Making

**DOI:** 10.1007/s11673-019-09945-x

**Published:** 2019-12-12

**Authors:** Craig Sinclair, Kate Gersbach, Michelle Hogan, Meredith Blake, Romola Bucks, Kirsten Auret, Josephine Clayton, Cameron Stewart, Sue Field, Helen Radoslovich, Meera Agar, Angelita Martini, Meredith Gresham, Kathy Williams, Sue Kurrle

**Affiliations:** 1 Rural Clinical School of Western Australia, University of Western Australia Albany Centre, 31 Stirling Terrace, Albany, Western Australia 6330 Australia; 2 Brightwater Group, 3/355 Scarborough Beach Road, Osborne Park, Western Australia 6017 Australia; 3 Helping Hand Aged Care, 34 Molesworth Street, North Adelaide, South Australia 5006 Australia; 4 UWA Law School, University of Western Australia, 35 Stirling Highway, Crawley, Western Australia 6009 Australia; 5 School of Psychological Science, University of Western Australia, 35 Stirling Highway, Crawley, Western Australia 6009 Australia; 6 HammondCare Centre for Learning & Research in Palliative Care and Northern Clinical, School, Greenwich Hospital, University of Sydney, Sydney, New South Wales 2065 Australia; 7 University of Sydney Law School, University of Sydney, Sydney, New South Wales 2006 Australia; 8 UWA Law School, University of Western Australia, Hackett Drive, Crawley, Western Australia 6009 Australia; 9 Improving Palliative, Aged and Chronic Care through Clinical Research and Translation, (IMPACCT), University of Technology Sydney, 235 Jones Street, Ultimo, New South Wales 2007 Australia; 10 HammondCare Dementia Centre, 97 River Road, Greenwich, New South Wales 2065 Australia; 11 Dementia Australia Consumer Dementia Network, National Office, 42 MacQuarie Street, Barton, Canberra, Australian Capital Territory 2600 Australia; 12 Northern Clinical School, University of Sydney, Sydney, New South Wales 2006 Australia

**Keywords:** Supported decision-making, Dementia, Substitute decision-making, Capacity, Phenomenology, Alzheimer’s

## Abstract

Supported decision-making has been promoted at a policy level and within international human rights treaties as a way of ensuring that people with disabilities enjoy the right to legal capacity on an equal basis with others. However, little is known about the practical issues associated with implementing supported decision-making, particularly in the context of dementia. This study aimed to understand the experiences of people with dementia and their family members with respect to decision-making and their views on supported decision-making. Thirty-six interviews (twenty-one dyadic and fifteen individual) were undertaken with fifty-seven participants (twenty-five people living with dementia and thirty-two family members) across three states in Australia. Interpretative Phenomenological Analysis (IPA) was used as the methodological approach, with relational autonomy as a theoretical perspective. We identified two overarching themes relating to participants’ experiences with decision-making: “the person in relationship over time” and “maintaining involvement.” Participant views on the practical issues associated with supported decision-making are addressed under the themes of “facilitating decision-making,” “supported decision-making arrangements,” “constraints on decision-making,” and “safeguarding decision-making.” While participants endorsed the principles of supported decision-making as part of their overarching strategy of “maintaining involvement” in decision-making, they recognized that progressive cognitive impairment meant that there was an inevitable transition toward greater involvement of, and reliance upon, others in decision-making. Social and contextual “constraints on decision-making” also impacted on the ability of people with dementia to maintain involvement. These themes inform our proposal for a “spectrum approach” to decision-making involvement among people living with dementia, along with recommendations for policy and practice to assist in the implementation of supported decision-making within this population.

**Electronic supplementary material:**

The online version of this article (10.1007/s11673-019-09945-x) contains supplementary material, which is available to authorized users.

## Introduction

The Convention on the Rights of Persons with Disabilities (CRPD) establishes a right to “legal capacity on an equal basis with others in all aspects of life” and an obligation on States Parties to provide citizens with access to support in exercising legal capacity (United Nations 2006). For people with cognitive disabilities (typically the result of intellectual disability, traumatic brain injury, mental illness, or dementia), who have traditionally been treated as subjects of protection (both within guardianship legislation and the parens patriae jurisdiction of the courts), this rights-based approach, centred on respect for the person’s will and preference, is paradigm shifting. While the CRPD has prompted significant debate about interpretation and obligations on signatory governments (McSherry 2012; Gooding 2015; Dawson 2015), there is broad agreement that it signals a need to question traditional practices relating to substitute decision-making on behalf of those with cognitive impairment.

Substitute decision-making is a term which has broadly been used to refer to the situation in which one person makes a decision on behalf of another. In many cases, substituted decisions are made on the basis of an “objective” determination of the person’s best interests,^1^ although other standards for substitute decision-making (e.g. substituted judgment) are established in some jurisdictions.^2^ The United Nations Committee on the Rights of Persons with Disabilities (2014) has identified substitute decision-making regimes as those in which

i) legal capacity is removed from the individual, even if this is in respect of a single decision;
ii) a substitute decision-maker can be appointed by someone other than the person concerned, and this can be done against the person’s will; and
iii) any decision made by a substitute decision-maker is based on what is believed to be in the person’s “best interests,” as opposed to being based on the person’s own will and preference.

Supported decision-making has been identified as an alternative to substitute decision-making and a “conceptual and practical bridge” (Gooding 2013, 432) which respects a person’s “will and preference,” while acknowledging the interdependence of decision-making. In this sense, supported decision-making has been understood as being grounded in the concept of relational autonomy (Series 2015), in which the human capacity for autonomy is developed, expressed, and always dependent on, supportive relationships of mutual recognition (Mackenzie 2008). Supported decision-making has been defined as “the process whereby a person with a disability is enabled to make and communicate decisions with respect to personal or legal matters” (United Nations Office of the High Commissioner on Human Rights 2009, 15), with support encompassing “both informal and formal support arrangements, of varying types and intensity” (United Nations Committee on the Rights of Persons with Disabilities 2014, 4). Browning has argued that supported decision-making can be understood in a range of ways, including as a process of supporting a person in making decisions (support *with* decision-making) and as a system or framework granting legal status to supportive relationships between people (Browning, Bigby, and Douglas 2014).

A recent scoping review of supported decision-making in Australia (Bigby et al. 2017) observed that existing literature in this area has taken a doctrinal or policy analysis approach (e.g. Australian Law Reform Commission 2014; Victorian Law Reform Commission 2012) or descriptive evaluation of pilot programs (ACT Disability, Aged and Carer Advocacy Service 2013, Wallace 2012), with a lack of empirical data on the characteristics of participants included or the effectiveness of resources or support arrangements. The small amount of empirical research undertaken in supported decision-making has focused on describing the experiences and perspectives of those in informal decision-making relationships and the practice of providing “support with decision-making,” as well as the broader processes involved in supporting the enactment of decisions made by a person with a cognitive disability (Knox, Douglas, and Bigby 2016). The focus has been predominantly on populations with intellectual disability and acquired brain injury (Bigby et al. 2017), with less attention given to people with dementia (Keeling 2016). This may be a product of the historical divide between the dementia sector and the broader disability advocacy movement. Although dementia advocacy by people living with dementia is now an established and growing movement (Shakespeare, Zeilig, and Mittler 2017), dementia was only globally recognized as a key human rights concern at the World Health Organization (WHO)’s First Ministerial Conference on Dementia in 2015, when Dementia Alliance International established the relevance of the CRPD as a rights-based instrument for people with dementia (World Health Organization 2015). This explicit recognition by the WHO confirms that dementia clearly falls within the scope of CRPD protections (Swaffer 2018).

Dementia has been defined as a syndrome caused by a range of medical conditions which have in common a chronic process of neurodegeneration leading to changes in a person’s memory, language, perception, attention, executive function, and/or emotion regulation (Australian Institute of Health and Welfare 2012). These changes in brain function are gradual and can fluctuate; however, dementia is a progressive and terminal condition, with no long-term effective therapy or treatment available at present. The prevalence of dementia is also increasing rapidly in Australia, driven in part by the ageing of the population and some increase in the rates of contributing risk factors, including cardiovascular disease (Australian Institute of Health and Welfare 2012, 2015).

The progressive cognitive impairment associated with dementia presents challenges for independent decision-making, as well as broader activities of daily living. Research exploring the perspectives of people living with dementia has shown that ongoing involvement in decision-making constitutes an important part of maintaining identity and positive coping with the condition (Fetherstonhaugh, Tarzia, and Nay 2013). However, research has also documented the use of substitute decision-making as the dementia progresses (Shanley et al. 2017). In a longitudinal study of everyday decision-making within families in which one person had dementia, participants described efforts to support the person in decision-making, while simultaneously navigating a transition towards substitute decision-making in response to disease progression (Samsi and Manthorpe 2013). These transitions in decision-making were experienced as stressful and associated with a risk of conflict, particularly among non-spousal family members. The relatively low uptake of legal instruments for nominating substitute decision-makers (White et al. 2014) means that in the context of conflict, the transition to substitute decision-making often also requires oversight by a tribunal or court, with formal guardianship being the usual result. Dementia can, thus, be understood as triggering the need for a range of decisions (e.g. financial, lifestyle, and healthcare) while simultaneously presenting challenges to the person with dementia and their family and close networks and the broader societal system of laws and policies in which they reside.

While there has been extensive research on the process and experience of substitute decision-making in dementia (Fetherstonhaugh et al. 2017; Givens et al. 2009; Lord, Livingston, and Cooper 2015), there has been relatively little research into the process of supporting a person’s ongoing involvement in decision-making. The current study aimed to understand the perspectives of people living with dementia and their family members with respect to key issues associated with supported decision-making. We focused particularly on:

1. the experiences and values of people living with dementia and their family members with respect to decision-making; and
2. the views of people living with dementia and their family members regarding the practical issues associated with implementing programmes or practices in the area of supported decision-making.

## Methods

This study is part of a larger project investigating community and professional attitudes with respect to supported decision-making in the context of dementia. This study takes a qualitative approach, drawing on a sample of semi-structured interviews undertaken with people living with dementia and family members of people with dementia, interviewed individually or in dyads. In-depth qualitative interviewing has been described as a way of documenting contexts and experiences but also a means of understanding the values and processes of making meaning that inform peoples’ practical approaches to decision-making, thus providing a bridge to ethical analysis and implications for policy and practice (Greenfield and Jensen 2010). The interviews addressed both the participants’ lived experiences of decision-making (including examples of “support with decision-making”) and their views on a formal mechanism for “supported decision-making.” The concept of relational autonomy (Series 2015; Nedelsky 1989) argues that decision-making reflects social contexts and interactions and is used as a theoretical perspective to understand participant descriptions of decision-making as being embedded within a social context. The study methods were approved by the University of Western Australia Human Research Ethics Committee (RA/4/1/8307).

### Study Design

Interpretative Phenomenological Analysis (IPA) was used as the methodological approach for this study (Smith, Flowers, and Larkin 2009). This qualitative methodology engages with the philosophical and practical challenges of interpreting the deeper meanings embedded in participant dialogue. The IPA approach has been employed in studies involving people living with dementia, in both individual (Fetherstonhaugh, Tarzia, and Nay 2013; Preston, Marshall, and Bucks 2007) and dyadic contexts (Merrick, Camic, and O’Shaughnessy 2016; Wawrziczny et al. 2016). The researchers acknowledged the existence of multiple social realities constructed between researchers and participants. Heidegger’s concept of “inter-subjectivity” was used to emphasize how interpretations of data are embedded in the socio-relational contexts in which they occur (Heidegger 1927).

### Sampling and Recruitment

The researchers recruited people who were formally diagnosed with dementia (at mild or moderate severity at the time of interview) and family members of people who were formally diagnosed with dementia. Participants were invited to be involved in individual interviews or dyadic interviews (person living with dementia and a close family member). In the case of the dyadic interviews, the person living with dementia and family member were in an existing care-partner relationship (n=15 spouse partners and n=6 parent–child dyads), with the family member typically the “primary caregiver.” The individual interviews included a sample of people living with dementia (n=4) and family members (n=11) who were associated with a non-participating person living with dementia (e.g. due to the person’s refusal [n=1], estrangement from the family member [n=1], severe cognitive impairment precluding participation [n=6], or having died [n=3]). If a person was involved in an individual interview, other members of their family were not approached to undertake an interview. Eligible participants were identified through partnerships with Dementia Australia, three large aged-care organizations across Western Australia, South Australia, and New South Wales, two multicultural seniors’ organizations and one community-based dementia respite facility. Table 1 shows characteristics of the study sample.

**Table 1:** Characteristics of interviews and participants

Characteristic	Participating people with dementia (n=25)	Participating family members (n=32)	Non-participating people with dementia (n=11)
Interview types			
Individual Interviews	4	11^(a)^	
Dyadic (spouse)	15	15	-
Dyadic (parent–child)	6	6	-
Age			
45 or younger	-	1	-
46-64	2	10	-
65-79	14	17	3
80 or older	10	4	4
Gender			
Male	9	13	7
Female	16	19	4
Place of birth			
Australia	19	21	3
Western Europe	4	4	1
Mediterranean Europe	-	5	6
South East Asia	-	1	-
South America	-	1	1
Africa	1	-	-
Location			
Metropolitan	15	20	5
Regional	10	12	2
Rural/Remote	-	-	-
Living arrangement			
Home	19	-	5
Residential care facility	6	-	2
Type of dementia			
Alzheimer’s	14	-	3
Vascular	1	-	2
Lewy body	1	-	1
Fronto-temporal	1	-	1
Mixed/other^(b)^	4	-	1
Unknown	4	-	3
Years since diagnosis			
Median (IQR)	5 (3–8)	-	4 (2.5–7)
Symptom severity (carer rated DSRS score)			
Average (range)	19.4 (6–36)	-	24.7 (12–32)

^a^ Individual family members of non-participating people with dementia were spouses (n=4), children (n=6), or parents (n=1). Some participating family members were estranged (n=1) or bereaved (n=3) at the time of the interview (age, location, living arrangement, and symptom severity scores are not reported in these cases).

^b^ Mixed/other dementia types included primary progressive aphasia (n=2), pressure hydrocephalus (n=1), Parkinson’s/Alzheimer’s, and dementia secondary to a traumatic brain injury.

Beginning with the assumption that every person is able to be supported to communicate their experience in some way, we employed a “process consent” approach to informed consent (Dewing 2007). Previous research has developed this approach in the context of participants with dementia and articulated five stages: preparation and background, establishing the basis for consent, obtaining initial consent, continual monitoring of ongoing consent, and feedback and support (McKeown et al. 2010). Potential participants were invited to nominate a time for one of the researchers to meet with them informally, build rapport, and explain more about the study. Wherever possible, this process was centred around the person living with dementia; however, in some cases other family members assisted in scheduling meetings. For people referred to the study by partner organizations, familiar service providers explained the project in broad terms and either introduced the researchers in person or obtained consent for the researchers to make contact. In a number of cases, a family member of the person living with dementia made initial contact with the researchers. The pre-interview informal meetings always involved the person with dementia (with or without their family member being present) and typically ran for twenty to sixty minutes. These meetings provided opportunities for the researcher to understand participants’ ways of communicating and their capacity to give informed consent and approaches to maximizing their involvement in the interview. The researchers generated memos about issues arising from these informal meetings and took detailed notes to inform any decisions about a person’s capacity to provide informed consent. Consent was obtained from participating family members for their own participation, and if required these family members also provided consent on behalf of the person living with dementia. In cases where substitute consent was obtained, assent was also obtained from the person living with dementia and maintained by checking with the person. If a person became confused or disoriented during the interview, the researcher paused to refocus the discussion, provided reminder cues about the study, rephrased questions with simplified language, and reintroduced themselves if needed as a way of maintaining the integrity of the consent process.

### Data Collection

Interviews were undertaken by three researchers (CS, KG, and MH), with training in psychology (CS) and nursing (KG, MH) respectively. The interviewers used a standardized form for documenting pre-interview and interview memos and met regularly during data collection and analysis to ensure a consistent and reflective approach. The interviews followed a discussion guide (see Appendix 1 in the Electronic Supplementary Material), using open-ended questions to initiate discussion about participants’ experiences in decision-making. The concepts of “supported” and “substitute” decision-making were explained in concrete, practical terms such as “having help to make your own decisions” and “having decisions made for you” respectively. The specific decisions explored in the interviews were directed by the participants and included healthcare, lifestyle, financial, and “everyday” decisions. Additional data included the Dementia Severity Rating Scale (Clark and Ewbank 1996), which was completed by a family member (n=32) or care provider (n=1) of the person with dementia, demographic information, and diagnostic history (type of dementia, time of diagnosis, and observing first symptoms). Researcher observations and memos were included as supporting data sources. Recruitment was ceased when it was agreed that a point of saturation had been reached in participant responses, along with a sufficient number of interviews being undertaken in key categories (individual and dyadic, regional and metropolitan, mild and moderate dementia, community and residential care dwelling) to ensure representation of different perspectives.

### Data Analysis

Interviews were audio-recorded and transcribed by a professional third-party company. The raw transcripts were processed by the relevant interviewer to check for accuracy and completeness and to ensure removal of any identifying names, places, or organizations. The processed transcripts were read closely prior to making margin notes, which included descriptive, conceptual, and linguistic observations (Smith, Flowers, and Larkin 2009), as well as “dyadic” observations (interactions between the two participants) where relevant. These notes informed the inductive process of assigning codes (units of meaning) and deriving emergent themes. Each code was defined and stored in a codebook, which was reviewed by the interviewers and organized into a preliminary framework. Each case was analyzed in detail, with creation of a summary which identified dominant themes arising in that interview. The process of deriving themes involved the interviewers and the broader research team, who discussed the data at a number of meetings. Emergent themes which were prevalent across the data set or which had specific explanatory power in relation to salient experiences were identified as “super-ordinate” themes (Smith, Flowers, and Larkin 2009).

## Results

Thirty-six interviews were undertaken across metropolitan and regional areas in three Australian states (Western Australia n=11, New South Wales n=15, South Australia n=10). The interviews lasted between 21 and 132 minutes (average 60.5 minutes). Analysis of the interview transcripts and researcher memos led to extraction of two themes relating to participants’ experiences and values in decision-making. We have labelled these “the person in relationship over time” and “maintaining involvement.” Participant views on the practical issues associated with implementing supported decision-making are described within four themes: “facilitating decision-making,” “supported decision-making arrangements,” “constraints on decision-making,” and “safeguarding decision-making.” Each theme is described and illustrated with exemplars.

### Experiences and Values in Decision-Making

#### The Person in Relationship Over Time

Participants described the importance of involvement in decision-making as a means of giving expression to a person’s unique identity and personhood. However, decision-making was not just “a single individual” at “a single moment” in time. Decision-making was described as being the product of multiple agents, linked through relationships and acting in roles over a period of time. In many cases the experience of dementia, which triggered a range of decisions and required significant support from close family members, prompted participants to describe the problem, and their responses, as being jointly owned.

… what we are faced with here is a problem that you and I have and you and I have to deal with it. So, it’s not the individual. It’s at least the individual and their carer. To take it further, it’s the family, the friends, the associates … all those people are somehow affected. (Tony, sixty-seven-year-old man living with dementia, interviewed alone)

Through this relational perspective, participants described how their own goals and values were shaped by considerations of the needs or desires of others. However, while decision-making was understood in a social context, many also described reduced access to social networks. One couple reported that many of their friends had “dropped off” since the diagnosis. For others the experience of stigma associated with their dementia left them less confident in their own abilities.

I’ve always been very confident and sure of myself but I’m not anymore … it’s just not there anymore. Probably because of other peoples’ reactions … (Josie, seventy-five-year-old woman living with dementia, interviewed with husband)

In rare cases this social exclusion was more explicit and directed by other family members. One woman described how her father’s feelings of shame associated with his wife’s dementia contributed to his tendency to exclude her from social situations and opportunities to engage with the broader community.

He’s just immaculate and everything has to be immaculate and Mum used to be immaculate and she’s not anymore. He can’t reconcile that. He thinks that the best way to deal with it is to keep it indoors, keep her inside. (Yulie, fifty-seven-year-old woman, daughter, interviewed alone)

In the context of declining social networks, there was an increased reliance on close, familiar people, usually close family members. One participant described how she observed that many people living with dementia lacked access to the normal social networks in which people explored decision-making options.

… because they’re not at work anymore to ask their workmates, and they’re not out with their mates anymore, which is the normal situations that you would sound off with other people. He really is stuck with me aren’t you? (Vivian, fifty-nine-year-old woman, wife, interviewed with husband).

Participants also described temporal aspects in decision-making, drawing on their histories and understandings of their likely future situations. For people with dementia, this temporal perspective was often influenced by difficulties remembering the past and a sense of foreboding about an uncertain and distressing future.

I sometimes think is it going to get worse? That’s the problem. Someone should be showering me or in there with me but is it going to be worse than it is now? I don’t know. No one can answer that one. (Barry, seventy-five-year-old man living with dementia, interviewed with wife)

For some, this prompted a desire to exert some control through planning for the future, while others preferred to maintain a focus on the present. These findings resonate with previous studies on how people with dementia and their family members understand their future in terms of a “not yet” horizon (Hellström and Torres 2016). Many participants also described significant transitions in key relationships and decision-making roles as the condition progressed, typically through close family members “moving in” or adopting substitute decision-making roles. A number of family members described how this was a difficult transition.

… becoming the parent of your parents is one of the hardest things that you have to do in your life. (Marika, seventy-seven-year-old (bereaved) daughter, interviewed alone)

These descriptions demonstrate relational and temporal dimensions of the decision-making context, reflecting the idea of the “person in relationship over time.”

#### Maintaining Involvement

A dominant theme across many interviews, voiced by people with dementia and family members, was the moral value of maintaining involvement of the person with dementia in decision-making for “as long as possible.” Participants also illustrated a range of practical benefits associated with this approach.

…there are occasions where [wife] makes decisions on my behalf but a fair percentage of those decisions she pays me the courtesy of what they are and why. I think that’s important … from the esteem side of things. (Tony, sixty-seven-year-old man living with dementia, interviewed alone)

One woman, a retired nurse, described how making her own decisions about her routine healthcare was an important part of feeling some sense of control in the context of having lost control in other areas.

I’ve lost so much. I’ve lost my confidence, my decision-making [has] gone through the roof … I was always on top of things. I’m not anymore and … my own health I feel I can still, especially something simple like a cough. (Josie, seventy-five-year-old woman living with dementia, interviewed with husband).

For another, the key thing was the feeling of being acknowledged through the decision-making process, rather than necessarily controlling the outcome of the decision.

It’s my life. I don’t mind if the decision doesn’t go my way, none of that matters. It’s to be acknowledged and recognized that you’re still a person and you’ve still got the ability to reflect what it is that I want, that I would like, and I’m quite happy to listen if there is to be another judgement I might shout a bit more, but… [aside to husband] we don’t have a problem, do we? (Sarah, seventy-one-year-old woman living with dementia, interviewed with husband).

These statements suggest that people with dementia felt that the efforts of family members to maintain their involvement in decision-making constituted an acknowledgement and contributed to their self-esteem and sense of personhood, resonating with previous research (Fetherstonhaugh, Tarzia, and Nay 2013).

Most family members corroborated these views, describing maintaining the person’s involvement in decision-making as a way of maximizing their wellbeing and quality of life. For some, this was described in moral terms, associated with respect for the enduring personhood of a person with dementia.

Right up until the last minute, they’re humans, they deserve to be respected the same way, they have rights … (Maisie, fifty-five-year-old (bereaved) daughter, interviewed alone)

Other benefits included enabling the person to maintain their habitual roles and responsibilities in relation to family members or other social networks. Finally, some also described their own distress or moral conflict associated with making substitute decisions and hoped to avoid this wherever possible.

… sometimes that’s quite complicated because if you’re a disability advocate as I have been for most of my working life the last thing you want to do is substitute decision-making and yet there are cases where I’ve come really close to that and I warned her of that. (Liesel, sixty-five-year-old woman, partner, interviewed alone)

While a majority of participants from both groups expressed support for maintaining involvement of the person with dementia in decision-making, there was also an acknowledgement that this involvement would inevitably change over time.

It comes back to identifying where that point in time is, where you become incapable of making decisions on your own … or even with assistance. (Tony, sixty-seven-year-old man living with dementia, interviewed alone)

Some people with dementia worried that they might make decisions that were inconsistent with their deeply-held values.

… if the disease damages my brain and I start to talk differently, that my guardians will implement what the real me, the undamaged me wants in place … (Vera, sixty-five-year-old woman living with dementia, interviewed alone)

Others expressed a preference to withdraw from active roles in decision-making, typically in cases in which there were trusted family members they could delegate to. This delegation to others was described with a sense of acceptance and relief.

As I say I’m pretty well off the hook for a lot of things, because my memory is mainly [pause] I discuss a lot of it with [daughter] and I rely on her for a lot of things… She took a lot of responsibility off my shoulders when she looks after things because I used to get worried because I was confused what I thought I might be doing wrong and she knew what I was trying to do. (Nerium, eighty-seven-year-old woman living with dementia, interviewed with daughter)

Family members also anticipated inevitable transitions in the person’s involvement in decision-making, often referencing the stage of the person’s dementia.

I think that [supported decision-making] will depend on the stage to be honest. That would be totally impossible now because there’s just not the cognition. It wouldn’t matter. I think definitely earlier on and after the doctors have deemed [husband] not competent, I still think there was probably another twelve months where we could have done that. (Vivian, fifty-nine-year-old woman, wife, interviewed with husband)

Family members tended to rationalize substitute decision-making with reference to the safety of the person with dementia or the need to make decisions that would manage their behaviour and keep other people safe.

… [making a substitute decision] didn’t worry me at all, because my main thing is he’s got to be feeling happy and contented and particularly safe, because he’s a very vulnerable man, although he wouldn’t like anyone to know that. (Barbara, eighty-five-year-old woman, mother, interviewed alone)

### Views on Supported Decision-Making

Participants’ views on supported decision-making are organized with reference to four themes: “facilitating decision-making,” “supported decision-making arrangements,” “constraints on decision-making,” and “safeguarding decision-making.” In responding to these questions, it was noticeable that some participants did not immediately perceive a difference between supported and substitute decision-making and required explanation and clarification of the concepts.

#### Facilitating Decision-Making

People with dementia and family members described a number of generic strategies aimed at facilitating the involvement of the person with dementia in decision-making. Taking time to explain concepts, being patient, repeating information as required, and not rushing the person with dementia were all seen as pivotal, with time constraints mentioned as a barrier to supported decision-making. Prompting was described as finding cues or giving increasing amounts of information which would function like “some little key,” unlocking a particular memory or understanding of a concept in order to orient a person to a decision or enable discussion about their wishes. People with dementia identified how close, familiar people who knew them well were often best placed to provide specific decision-making support in efficient ways.

… [my] partner’s very good at knowing what my preferences are to start with and for example going out to dinner … She made the decision to narrow it down to two or three venues that I would function with. Offered a narrow choice which fitted in with what I would have gone with anyway. But it saved the problems of having to dismiss other options … (David, sixty-three-year-old man living with dementia, interviewed alone)

Interpreting or “translating” information into simpler concepts was frequently mentioned, as well as the use of memory prompts or reorienting the person to the conversation. Others spoke of looking for repeated statements of the person’s preferences to confirm their understanding and the reliability of their request, particularly for more “serious” decisions.

The most important thing that … [friend] did was recognizing at that stage there was [sic] periods of lucidity and … to use that, not just once but three or four times to confirm and confirm and confirm. (Vivian, fifty-nine-year-old woman, wife, interviewed with husband)

Family members described how their techniques were sensitive not only to the “stage” of a person’s dementia, but also to the type of cognitive impairment that they perceived the person to be experiencing (see table 2), as well as the broader social contexts which might also constitute barriers to decision-making (see table 3). Identification of these factors was associated with awareness of the person’s specific vulnerabilities as well as spared abilities and suggested a range of more targeted strategies for providing decision-making support. While these strategies were often effective, people with dementia and family members also identified practical and emotional difficulties associated with facilitating decision-making. Chris described a sense of distress associated with always having to be “the no person” for his wife, who had dementia. Josie described the experience of being “bombarded” with assistance from family members and the need to get the amount of support just right.

… you feel as though you’re being bombarded. If somebody’s trying to tell you something like [husband]’s been trying to tell me something sometimes and he says, “But I mean this, this is what I’m meaning,” and not catching on and then I lose it because it’s just not working. (Josie, seventy-seven-year-old woman living with dementia, interviewed alone)

**Table 2:** Clusters of cognitive impairment (as identified by participants) and strategies to facilitate decision-making

Cognitive Impairment	Strategies	Exemplars
Difficulty retaining information	• Repeating information • Alternative approaches to access memories	“I may have to have that conversation with mum many times, and that can be challenging, but when I’m in the moment with her she understands it.” (Gloria, fifty-eight-year-old woman, daughter) “So if you describe a picture then [partner] remembers things … but the way she learns and perceives the world is very different from many people …” (Liesel, sixty-five-year-old woman, partner)
Difficulty understanding or weighing up multiple options	• Translating jargon • Simplifying concepts • Presenting a reduced number of options • One topic at a time	“When we got to [completing advance care] directives, the lawyer was a bit concerned that he may not understand … but when I said, look [lawyer] is talking about when you’re ill and you’re in hospital and the doctors want to give you perhaps oxygen or even put you on a drip and all sorts of things, what do you feel about this? He said no, he doesn’t want any messing about.” (Barbara, eighty-five-year-old woman, mother)
Vulnerability to suggestion	• Safeguarding decision-making process by knowing the person well • Transparent communication	“Unless you really get to know that person and know who they are as a person, you’re not going to notice anything different … whoever makes or becomes an assistant decision-maker needs to be able to spend time with that person as in a live-in situation …” (Terri, fifty-eight-year-old woman, daughter) “Family [members] have got to be kept in the loop communication-wise. They have got to, be it a written report or a verbal report …” (Tony, sixty-seven-year-old man, living with dementia)
Impulsivity	• Managing risks	“I think you have to let people do it and perhaps take away as many of the risks as possible and let them still do it. And you’re dancing around the edges trying to keep him safe …” (Barbara, eighty-five-year-old woman, mother)
Difficulty communicating choices	• Assistive technologies • Alternate media (e.g. visual, tactile) • Photographs and cards with significant people or regularly used words	“The fact that someone can’t easily express what they want or what they think should not be a barrier to their inclusion or participation so it’s about reasonable adjustment. To have a note taker is obviously easily enough done.” (Liesel, sixty-five-year-old woman, partner) “I go ‘can you show me? If you can’t show me, write it’.” (Richard, seventy-five-year-old man, husband)

**Table 3:** Social contexts constituting barriers to decision-making involvement (as identified by participants) and strategies to facilitate decision-making.

Barrier	Strategies	Exemplars
Social isolation	• Support groups • Circles of support	“… in this area we’ve created a support group, peer support group, and within that we have family, friends, neighbours, people with dementia … gradually trying to break down that isolation.” (Liesel, sixty-five-year-old woman, partner) “… [person with dementia] has a circle of friends that help him make decisions. They have a sort of a conference type situation, or he’ll go to one of them and ask.” (Barbara, eighty-five-year-old woman, mother)
Lack of suitable options	• Tailoring individualized approaches to traditional service models	“Because by respite what they meant was short term stay in residential aged care and they [dementia area advisers] were insisting on it because they know that you can’t keep going by yourself and you can’t, and also because they know that there are not alternatives easily available … [so] we’re organizing for her to go and spend some time in [city] with her family and I’ll have some time at home alone and a care worker will go with her and that’ll be good …” (Liesel, sixty-five-year-old woman, partner)
Attitudes of service providers	• Advocacy	“I had to reprimand one. [Support worker] actually said that [person with dementia] couldn’t do something and he had to go and do something else. Unfortunately, I heard and said do you know [person with dementia] pays your wages? It’s his house and he’s a grown man. He apologized.” (Barbara, eighty-five-year-old woman, mother)
Opposition from other family members	• Independent and credible health professionals • Independent “ombudsman”	“I believe it should be the role of the GP to instigate that conversation with the person living with dementia and then with their partner or family or whoever, to be made aware of the benefits of supportive decision-making …” (Maisie, fifty-five-year-old woman, daughter)

#### Supported Decision-Making Arrangements

In response to questions about how supported decision-making might be implemented in their own personal situations, participants gave a variety of responses. It was quite common for participants to interpret the idea of supported decision-making with some initial alarm, associated with the assumption that the support would come from an “outsider,” intruding on existing relationships. This response was particularly common among participants interviewed with family members present. Close family members and friends were valued in terms of having “deep” knowledge of the person with long-term relationships that preceded the dementia diagnosis. They were perceived to be more trustworthy. One man described how he would be more likely to trust a supporter from within his family network than a person who was employed to undertake this role.

I think that’s one of the first things I would be wary of, the person who was … unless it was my mother or sister or [wife] or somebody like that, who knows me and I have got complete faith in. If it was someone who [was] employed … I don’t know who they are. I don’t know what they are like. So, you don’t give your innermost thoughts to somebody who you don’t know what they are going to do with them. (Derek, seventy-two-year-old man living with dementia, interviewed with wife)

Despite these established relationships and associated deep knowledge of the person with dementia, there were some issues identified with involvement of close family and friends in supported decision-making. These mostly related to their lack of experience or specific knowledge with respect to complex systems (e.g. aged care or social services). This was illustrated by instances in which people with dementia turned to specific family members or friends with relevant professional experience (e.g. health professionals) for help in making decisions. Other difficulties associated with utilizing close family members or friends in supported decision-making were competing priorities or conflicts of interest, disputes with other family members, and the family member’s own experiences of illness, functional decline, or burnout in existing caring roles. In the context of these limitations, external supporters or facilitators were seen to have value in terms of providing expertise relevant to a decision and being independent from family relationships and hence able to provide more objective input.

… on balance, a little bit of both is probably what you need. The objectivity and the guidance … (Daniel, seventy-five-year-old man, husband, interviewed with wife)

There were some interesting differences among participants living with dementia who were interviewed alone. David described how his sister, who was also diagnosed with dementia, had experienced her children “taking over,” making decisions that he felt were driven by their own self-interest. Based on this, he described the value of an outside, impartial third party in protecting the person with dementia from self-interested decisions made by family members.

… if there was a third party involved in effect, independent of them to consider both sides of what’s involved in the decision, the outcomes may be different and may be better for the person in care. (David, sixty-three-year-old man living with dementia, interviewed alone)

Another participant living with dementia felt that independent supported decision-making “facilitators” could bring specialist knowledge pertinent to the issues and decisions facing the person living with dementia, particularly in areas that a lay person would not be conversant with.

So, highly qualified people who can make a difference and then can comment intelligently on the problem at hand. If they don’t understand it, they will seek it out, seek the answer out. (Tony, sixty-seven-year-old man living with dementia, interviewed alone)

Interestingly these two participants did not immediately nominate the “supporter” role to family members, although both described accessing significant day-to-day support in decision-making from their spouses and social networks. They both saw that this role could be aligned with that of a “dementia key worker,” a support and service navigator role that they were both familiar with (cf. Renehan, Goeman, and Koch 2017). Both described the need for ongoing assessment of decision-making capacity and oversight of supported decision-making by a pseudo-ombudsman position. One of these participants acknowledged that family members would probably object to utilizing “outsiders” in this role.

… there would be, I’m sure, family and carer objections all over the place. “They can’t do it as good as us. They don’t know him as well as we do” … (Tony, sixty-seven-year-old man living with dementia, interviewed alone)

#### Constraints on Decision-Making

In describing the possibilities for facilitating the involvement of people with dementia in decision-making, both groups of participants identified a range of constraints, which they saw as impacting on the number or types of choices available or the voluntariness of a person to freely choose one option over another. David, a man living with dementia, identified difficulties in taking the necessary time to make decisions when professionals required them to be made quickly, on a “corporate timeframe.” For many participants, financial constraints impacted on the options that were available and influenced healthcare and other lifestyle decision-making. In some cases, specific cultural beliefs and community expectations also appeared to constrain decision-making.

… my mother had to pick one of [daughter and son] to be the decision-maker. Her heart would be with me but the pressure for her culturally would be to choose my brother because he’s the bloke. (Yulie, fifty-seven-year-old woman, daughter, interviewed alone)

One woman living with dementia identified the potential difficulties that might arise in maintaining a supporter if she expressed a view that disagreed with what the supporter felt. She saw that this would lead to a risk of losing their assistance or endangering the relationship.

I would feel if I don’t more or less do what they say, or they suggest, even though they know how I feel, they might just say “okay well you do what you want to do” and then they leave. (Ruby, sixty-seven-year-old woman living with dementia, interviewed with husband)

#### Safeguarding Decision-Making

In discussions about safeguarding supported decision-making processes, many participants reiterated the importance of involving close, familiar people (typically family members). These people were often described as being more trusted because of their relational connection and their investment in “doing the right thing” by the person. For others, the value in involving these people was that they would be more likely to understand the person’s perspectives and values and be able to use this knowledge in assessing advice coming from other sources.

If you get someone from outside who’s looking at the circumstances and they come they say, okay I think it would be a very good idea, this is what we could do and how we could do it, but then have it modified or altered by, be it [daughter] or whoever, who looks at me from the heart, rather than from the reality—they know me from the heart, rather than the reality. (Sarah, seventy-one-year-old woman living with dementia, interviewed with husband)

However, alongside this was the recognition that close family members might also be a source of undue influence or abuse, even when purportedly providing decision-making support.

That’s really, a real bucket of worms that one … I can equally see a case where the carer, for argument’s sake, has a mercenary viewpoint and arranges to suit his [sic] own sort of end … it’s fraught with danger that one, from both sides. It’s one that’s easily abused. (Chris, seventy-one-year-old man, husband, interviewed with wife)

In this context, some participants identified the possibility of involving independent third parties, either in professional roles or more informally through community support circles.

… it’d be nice if there was also an oversight in that as well. Like an ombudsman type system where those decision-making processes could be questioned if need be or reviewed so that it’s not just … becoming a token system … (David, sixty-three-year-old man living with dementia, interviewed alone)

Many people noted how decision-making in the context of dementia was potentially a source of conflict due to different beliefs, values systems, and “realities” experienced by different people in the person’s family and social networks. For some this led to a “collision of values” that was expressed in conflict around decision-making, and resultant communication difficulties. In some cases, these conflicts were described as being caused by the progression of dementia leading to transitions in decision-making (greater involvement by one family member), which were interpreted by other family members (who had not been as involved in the person’s care) as “taking over.” Others described histories of problematic relationships with other family members and how this impacted negatively on establishing collaborative decision-making processes. To address these issues, or pre-empt future problems, participants felt that proactive communication by the supporter would help other family members understand the process and would assist in keeping them informed and involved where possible. One respondent, who experienced conflict within her family associated with her advocating for maintaining her father’s role in decision-making, described how credible, independent professionals (such as their general practitioner) could have promoted supported decision-making principles and encouraged open discussion within the person’s family, beginning at the time of diagnosis.

… if it was made like a protocol, like a guideline, I think people then do [adopt a supportive approach], but for me for instance if I come and said look “I’ve been reading and I think this and this”—well “so what?” They [other family members] don’t really listen to you or they think that’s your opinion. (Maisie, fifty-five-year-old (bereaved) daughter, interviewed alone)

## Discussion

This paper describes the experiences and values of people with dementia and their family members with respect to decision-making and their views on supported decision-making. While many participants welcomed the principles of supported decision-making and situated these within their existing philosophy of maintaining involvement of people with dementia in decision-making for as long as possible, practical challenges were identified. In other cases, participants found it difficult to discern between the approaches of “supported” and “substitute” decision-making. Interpreting these views provides insights into the alignment between current supported decision-making policy discourse and the practical understandings and experiences of people with dementia and their family members. Successful implementation of supported decision-making in the context of dementia will require policies that align discourses, services, and resources with these practical experiences and identified needs in order to generate clear and consistent messaging to professionals and the community. We propose a number of strategies within an overarching “spectrum model” of supported decision-making which is applicable for people with dementia and conclude with discussion of the strengths and limitations of the study and implications for policy and practice.

There is a dynamic tension between the moral value of “maintaining involvement” and the acceptance of a psychosocial process of transition towards increasing reliance on others in decision-making. This tension is reminiscent of Fetherstonhaugh’s (2013) depiction of people with dementia simultaneously “holding on” and “letting go.” This was observable both for people with dementia and their family members, with the transition being described as a difficult and uncertain process. The recognition of a process of transition between independent, supported, and substitute decision-making suggests a “spectrum” of decision-making involvement, which could vary depending on the decision and will likely evolve over the disease trajectory (Samsi and Manthorpe 2013; Sinclair et al. 2018). This is particularly relevant in dementia due to the gradually progressive nature of the cognitive impairment over time. However, in addition to biomedical aspects of the disease, social and contextual factors were also relevant, including social isolation or exclusion, lack of suitable options, and unhelpful attitudes or opposition from family members or service providers. In some cases, these factors appeared to exacerbate existing cognitive impairments, whilst in others they constituted direct barriers to decision-making involvement. Understanding and facilitating the strategies already adopted by people with dementia and their family members to address these issues (see table 3) while providing assistance at transition points in decision-making involvement may assist in broader implementation of supported decision-making.

People living with dementia described the importance of trusted relationships with close family members in facilitating their decision-making. These people were seen as being best equipped to assist the person with dementia in understanding issues and communicating their will and preference to others when needed. People with dementia expressed values that considered the needs and situations of other family members and a desire to maintain relationships and relational roles. Many were suspicious about the prospect of “outsider” involvement in their everyday decision-making and preferred to delegate decisions to trusted family members where this was necessary. However, there was also recognition of the potential for family members to abuse positions of trust and the role for independent professionals in mentoring or overseeing supported decision-making. These findings suggest the importance of professionals being sensitive to a person’s relational histories and other relevant social factors when attempting to understand why a person might want to make or delegate certain decisions (Ho 2008; O'Connor 2010). While this may be complicated in situations where abuse is suspected, the risk of viewing decision-making through an individualistic lens is that it neglects peoples’ true motivations and experiences. At a broader level, it raises questions about the prevailing discourse of supported decision-making, which seems to foreground “individual autonomy” and “rights of people with disabilities,” while simultaneously invoking “relational autonomy” in decision-making as the means of achieving this, often without a clear articulation of how these two concepts interact (Series 2015) or how this might be experienced by family members. It may be that understanding a person’s values, social situation, and trusted supporters, and providing education, support, and mentorship for the “relational decision-making unit” will be more effective in harnessing the cooperation of existing support networks than a “rights-based discourse” grounded in individualist notions of “me” and “my decisions.” A growing recognition of the importance of informal support networks and the growth of psychosocial interventions for dyads in which one person has dementia (Whitlatch et al. 2006) might be augmented by inclusion of education and resources for these people to enable informal supported decision-making, ideally as soon as possible following diagnosis.

### A Spectrum Model for Supported Decision-Making in Dementia

**Fig. 1:**
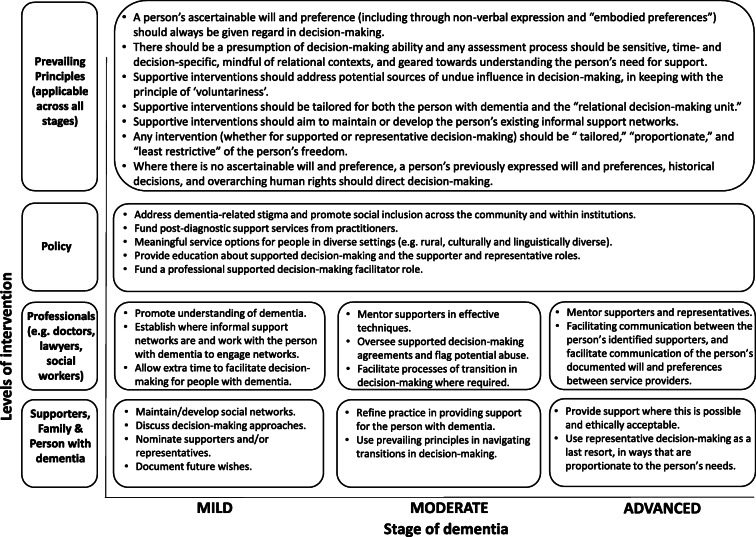
The strategies that might be implemented in a “spectrum model” of supported decision-making in dementia. This model suggests an emphasis on different strategies at different stages of the condition. The model presumes a timely diagnosis of dementia during the “mild” stage.

Based on participant responses we suggest a range of levels and types of support will be required, matched to peoples’ situations and the nature and extent of their cognitive impairment. As cognitive impairment progresses, this “spectrum model” would require ongoing formal (e.g. professional) and informal (e.g. family member) monitoring underpinned by a (rebuttable) presumption of decision-making being directed by the person’s will and preference and a least restrictive approach to supportive interventions (see figure 1). In the early stage of dementia, a range of supports should be offered, aimed at reinforcing existing support networks and addressing social isolation or exclusion, with specific resources tailored to the wants and needs of the person. As the condition progresses, the nature of the required support may change and in some cases the person’s needs may move across the spectrum towards a point where “support with decision-making” is not practicable (e.g. it is difficult or impossible to elicit a person’s will and preference) or is ethically untenable (e.g. efforts to support a person’s will and preference places the person or others at manifest and unreasonable risk of harm). At this point, we imagine that supporters would need to take a more active role, striking a balance between eliciting and acknowledging the person’s current will and preference where possible while also respecting their previously established will and preferences (e.g. via advance directives), along with a prevailing respect for their enduring human rights, of which “legal capacity” is just one (United Nations 2006). This approach is aligned with the “representative” role articulated in recent law reform agency reports (Australian Law Reform Commission 2014; New South Wales Law Reform Commission 2018). Importantly, the role would be a departure from conventional “substitute decision-making” approaches, in which objective determination of the person’s best interests directs decision-making. The “stepped” approach that incorporates both a “supporter” and a “representative” role enables flexibility and proportionate responses to disease progression or situational demands. This approach is reminiscent of the “stepped model” of supported decision-making articulated in previous supported decision-making pilots (Office of the Public Advocate (South Australia) 2013); however, in the context of dementia the transitions over time are likely to be towards increasing support rather than eventual independent decision-making as originally conceptualized in the “stepped model.”

The challenges in implementing a stepped approach in dementia would be in navigating the gradual and fluctuating but progressive transitions in decision-making. In a previous paper focusing on couples’ experiences with decision-making, we have illustrated how these transitions are neither linear nor predictable, with “independent,” “joint,” “supported,” and “substitute” decision-making often intertwining in everyday life (Sinclair et al. 2018). Related to this, it may be that the resourcing requirements for support with decision-making do not necessarily increase across the course of the disease. The psychosocial impacts of a dementia diagnosis on the person and their family unit and the complexity of social, relational, and role transitions occurring in the early stages of the disease may mean that support needs for some people will be greatest in the early or middle stages of dementia. Furthermore, the degree of support required may fluctuate depending on the nature and complexity of the decision being made. Research has also shown that diagnosis is not always timely and is sometimes not fully disclosed to the person with the condition (van den Dungen et al. 2014). The clinical syndrome of “anosognosia” (lack of self-awareness of one’s own disability) also occurs in dementia (Lehrner et al. 2015) and may result in people rejecting support from others in decision-making. Clinical practice in this area will require a high level of assessment and communication skills, with attitudinal shifts also being required in many cases. Acute medical crises will likely present additional challenges, particularly a lack of time. We propose that a number of principles can guide professional and community practice in navigating transitions in decision-making (see figure 1). At the top of the list would be the will and preference of the person with dementia, including their preferred level of involvement in the decision at hand. The person’s access to formal or informal support and ability to understand issues relevant to the decision (in the context of all available supports) should also be considered, along with prevailing principles of voluntariness, proportionality, and least restrictive practice in both supported or “representative” decision-making. In light of the CRPD principles and the importance placed upon inclusion, participation, and autonomy we would argue that a prevailing ethos supporting positive risk-taking and “dignity of risk” is ethically justifiable, particularly where these risks are taken in the interest of obtaining or maintaining benefits that are in line with the person’s values, as endorsed through their statements and actions over the course of their life (Phillips and Wendler 2015).

The theoretical perspective of relational autonomy explains how one person’s autonomy can be developed and expressed through close, sustained relationships and the mutual recognition which this entails (Mackenzie 2008). This perspective is important in the effective implementation of supported decision-making, as the participants consistently identified their preferences for shared decision-making processes and mutually endorsed outcomes. However, we also note that one interpretation of relational autonomy (the “strong” interpretation) which entirely subsumes the individual into a collective (or another) is inconsistent with the views of many of the participants and, we would argue, untenable in practice (e.g. Oshana 1998). This “strong” interpretation is evidenced by the use of the concept of “total support” in connection with supported decision-making (United Nations 2007). Total support can be defined as a decision made notionally “by” a person with a disability, where the decision is in fact made by a supporter in circumstances where the decision-maker is unable to communicate any decision or preference. A strong interpretation of relational autonomy suggests that “total support” is an authentic exercise of autonomy by the person being supported. This not only creates conceptual confusion, but also, significantly, a “legal fiction” in suggesting that the decision has been made by the person with a disability in circumstances where he or she is not actually capable of exercising autonomy in any meaningful way (Burch et al. 2014). To label this “total support” may validate the actions of supporters who are in fact unjustifiably overstepping the supporter role. Our research shows that people with dementia want support to maintain involvement in decision-making *up to a point* but are also mindful of scenarios in which they were unable to make decisions, even with all available assistance. We argue that strong, enduring relationships can provide a context for a person’s supported decision-making, to the extent that such support assists the person in identifying themselves as “able and authorized” to make (and own) the decision, even where the decision itself is then enacted by others. This is consistent with a “weak” interpretation of relational autonomy which recognizes the social context as an enabler, rather than the dominant factor, in the process of decision-making (Mackenzie 2008; Pritchard-Jones 2017). Where the person’s sense of self-identification as the decision-maker is not possible, we suggest that the term “representative” more accurately describes the role of others in the decision-making.

#### Implications

Based on the responses of people with dementia and their family members, we suggest a number of implications for the further development of supported decision-making in the context of dementia. These include i) the provision of a formal framework for supported decision-making, ii) development of a professional role to provide mentorship, oversight of, and advocacy for supported decision-making arrangements, and iii) broad-scale advocacy, education, and community development to address the social and contextual barriers faced by people with dementia in decision-making.

##### A formal framework for supported decision-making

Based on participant responses, we are calling for a formal framework for supported decision-making agreements. While some people with dementia will prefer not to access such an agreement, a formalized framework will assist in clarifying the supporter role, enabling a clear process for the safe sharing of personal information associated with decision-making and providing for ongoing mentorship and oversight of supporters. While supported decision-making has promise in assisting people with dementia to maintain involvement in decision-making, there is a limit on the extent to which some people will be involved, particularly those with advanced dementia. A substantial group of people with dementia express a preference to delegate some or all of their decision-making to trusted others through formal and informal mechanisms. Formal frameworks should articulate how the supporter and representative role can exist together and the process by which the representative role can be invoked as a last resort—for the shortest time possible and subject to independent review. Where representative decision-making is used, the standard for this approach should require representatives enact decisions that foreground the person’s currently ascertainable will and preferences while giving consideration to their previously expressed wishes and their enduring human rights, including safety and social inclusion. Supporters, representatives, and professionals will need assistance and resources in determining at what point the “support with decision-making” approach is no longer possible, but it is inevitable that formal capacity assessment must play a role in this determination, particularly for “high stakes” decisions with legal implications. Educational resources will also need to address the issue of how a person’s will and preferences can be ascertained when there is no record of them and the person is unable to communicate.

##### Development of a professional supported decision-making role

Participants recognized their need for greater understanding of dementia and expert assistance with more complex decisions, particularly in situations where the person with dementia was vulnerable to abuse or other harms (e.g. physical or financial risk) associated with decision-making. The paradigm-shifting nature of supported decision-making further suggests that its implementation in the professional sphere will take time and require significant education and attitudinal change. One approach is developing a specific professional role within services—bringing expertise to engage with people living with dementia and their chosen supporters as a facilitator or mentor—while also liaising with and supporting other professionals regarding the complexities of supported decision-making and adopting an advocacy and “champion” role in the professional sphere. Such a role would require an understanding of the existing environment of available services and resources. By connecting such professionals with people with dementia early in the course of the illness, there will be a greater likelihood of establishing a trusted relationship, which might alleviate tensions associated with “outsider” involvement. This ongoing involvement might also assist in pre-empting situations that may lead to abuse within family relationships. The role should adopt an overarching commitment to supporting the relational decision-making unit and should aim to build capacity within existing informal support networks wherever possible, rather than directly providing resource-intensive “decision support.” Given the complexity of this role, the intensity of facilitation or mentorship that might be required, the likely requirement for acute responses to after-hours “crises,” and the projected increase in the population of people living with dementia, such a role would require significant resources and funding. In some jurisdictions this has been addressed through the development of a cohort of volunteers (Office of the Public Advocate (Victoria) 2013).

##### Advocacy, education, and community development to address social and contextual barriers

The repeated observation of significant social and contextual barriers (e.g. social isolation or exclusion, lack of suitable options, and unhelpful attitudes or opposition from family members or service providers) to the involvement of people with dementia in decision-making suggests the need for advocacy, education, and community development. In Australia, this is currently being addressed to some extent by a range of advocacy organizations and service providers. This current activity could be augmented through an explicit focus on the key principles underpinning the CRPD and provision of education resources for such organizations to assist staff in articulating how these principles would be applied in the context of dementia. While such interventions can be costly, diffuse, and difficult to evaluate, any benefits associated with raising awareness of supported decision-making will likely also advance related priority areas such as promoting inclusion of older people in society and addressing stigma associated with dementia. Addressing the constraints associated with a lack of choice in service provision or social isolation among people with dementia may require a more structured community development approach. Emerging consumer-led peer support and advocacy networks (e.g. Keyes et al. 2016), “dementia-friendly community” initiatives, and local action groups may provide cost-effective opportunities for such community-based intervention to develop and reach new populations.

#### Limitations

This study had some limitations, including sampling constraints and issues associated with the study procedure. The sampling of participants for dyadic interviews was limited to those in family relationships (spouse partners and parent-child dyads). This may have contributed to the tendency for participants to express a preference for supported decision-making occurring within close and trusted family relationships. While we had initially envisioned recruiting a sample of people in “other” relationships (e.g. more distant family, friends, or neighbours) this group was difficult to recruit. It was also difficult to gain access to people with dementia who lacked any close relationships; this was perhaps a result of the recruitment methods. Understanding the experiences of these groups, who are arguably more vulnerable, is a direction for future research. This study was limited to individual and dyadic interviews and did not undertake broader interviewing or observation to assess the perspectives of other family members associated with the person with dementia or the role of the broader “family system” in influencing the decision-making context (Purves and Perry 2009). Future research might address this limitation by interviewing multiple family members associated with a person with dementia in order to understand the role of different perspectives and relationships on the experience of decision-making.

In terms of the study procedure, there are limitations inherent in the interviewing paradigm, which relies on recall of prior experiences and receptive and expressive communication capacities and by its nature can exclude some people. This was addressed where possible; for example, one participant with expressive aphasia communicated in short phrases, supplementing this with written responses on a notepad where needed. Furthermore, while the researchers were directed by participants in terms of the types of decisions they wished to discuss, it is possible that the interview approach biased the discussion towards “high-stakes” decisions and downplayed the (also important) dynamics of “everyday” decision-making, including pre-reflective expressions of a person’s preferences. Further work might specifically explore how supported decision-making practice could engage with these “embodied preferences” as a means of acknowledging the enduring personhood and agency of people with even very advanced dementia (Kontos, Miller, and Kontos 2017; Grigorovich and Kontos 2018). Such work will inevitably encounter interpretative challenges (e.g. Series 2015) and tensions associated with pre-reflective embodied preferences that appear to conflict with a person’s previously expressed will (e.g. Dworkin 1986; Dresser 1995). Resolution of these tensions may come through recognition of different thresholds of clarity, consistency, and reliance on others in the expression of will and preference for routine, everyday decisions, as opposed to decisions with significant legal implications.

## Conclusion

Dementia is a condition resulting in gradual and progressive decline, but with an unpredictable course. This, along with the pluralism in peoples’ values, preferences for involvement in decision-making, family support structures, and social networks indicates that no single type of supported decision-making will be suitable for all people living with dementia. We propose a spectrum model of supported decision-making which incorporates both a formal framework for “supporters” and recourse to a “representative” role as a last resort. Implementation of supported decision-making will need to harness a person’s existing support networks and supplement this where needed with external facilitation. Educating service providers and the broader community while addressing the broader social and contextual barriers to decision-making involvement experienced by people with dementia will be a critical component of any policy aimed at promoting supported decision-making for this population.

## Electronic supplementary material

Online Resource 1 (PDF 65.1 kb)

## Footnotes

1 For example, as legislated in *Guardianship Act 1987* (NSW) s4(a).

2 For example, *Guardianship and Administration Act 1993* (SA) s5(a).
