# Supplementary material for: “A Real Bucket of Worms”: Views of People Living with Dementia and Family Members on Supported Decision-Making
Source: J Bioeth Inq. 2019 Dec 12;16(4):587–608. doi: 10.1007/s11673-019-09945-x (PMC6937221; doi:10.1007/s11673-019-09945-x)
Supplement: Supplementary file 1 — (PDF 65.1 kb) [file 11673_2019_9945_MOESM1_ESM.pdf]

1A. INTERVIEW DISCUSSION GUIDE – Dyad or individual person with dementia

We are interested in understanding more about how people make decisions about their lives and plan for the future… for things like healthcare or personal matters like where they live… We want to know how people make these decisions and whether the laws for this decision-making are working…

Experiences with current legal approaches to decision-making

- *Can you tell me about your experiences making decisions over the last few years?*
- *Have there been any times where people have made decisions for you? ... What happened? ... Looking back, how did that feel?*
- *Have there been times where people have helped you to make your own decisions? ... What happened? ... What did they do to help? ... Looking back, how did that feel?*

Understanding of current legal approaches to decision-making

- *What happens if a person can't make an important decision for themselves?*
- *If a person can't make a decision for themselves, the law allows someone to be appointed, to make the decision for them, usually a family member or guardian. How do you feel about this?*

Perspectives on supported decision-making

- *Some people think we should have a new type of law, that lets people choose someone to help them make decisions, rather than making the decision for them. How do you feel about this?*
- *If there was a law that let you choose someone to help you in making decisions for yourself, would you nominate a person? ... If so, why? ... If not, why not?*
- *If you were going to choose someone to help you in making decisions, how would you choose? ... What could they do to help?*
- *What types of decisions would be good to have help with? ... What sort of help would be most useful?*
- *Are there any problems you can think of with having help to make decisions, rather than doing it on your own or someone else making the decision*
- *If people were allowed to choose someone to help them make decisions, what advice would you give, to help make it work better?*

Supported decision-making scenarios

- *Some people think that if people choose someone to help them make decisions, the helper might end up taking over. What do you think about this? ... What could be done?*
- *Some people think that if a person has problems with their thinking or memory, that it might be safer for someone else to make decisions for the person. What do you think about this?*
- *Some people think that if people choose someone to help them make decisions, there might be disagreements or arguments. What do you think about this? ... What could be done?*

1B. INTERVIEW DISCUSSION GUIDE – Carers of people living with dementia

We are interested in understanding more about how people make decisions about their lives and plan for the future… for things like healthcare or personal matters like where they live… We want to know how people make these decisions and whether the laws for this decision-making are working…

Experiences with current legal approaches to decision-making

- *Can you tell me about your experiences with decisions involving [name of person with dementia] over the last few years?*
- *Have there been times where you have made decisions for [name of person with dementia]? ... What happened? ... Looking back, how did that feel?*
- *Have there been times where you have helped [person with dementia] make their own decisions? ... What happened? ... What did you do to help? ... How well do you think it worked? ... Looking back, how did that feel?*

Understanding of current legal approaches to decision-making

- *What happens if a person can't make an important decision for themselves?*
- *If a person can't make a decision for themselves, the law allows someone to be appointed, to make the decision for them, usually a family member or guardian. How do you feel about this?*

Perspectives on supported decision-making

- *Some people think we should have a new type of law, that lets people choose someone to help them make decisions, rather than making the decision for them. How do you feel about this? ... How would this work for a person living with dementia? ... How would this work for [name of person with dementia]*
- *Are there any problems you can think of with people having help to make decisions, rather than doing it on their own or someone else making the decision?*
- *If there was a process in which people were allowed to choose someone to help them make decisions, what advice would you give, to help make it work better for people living with dementia?*
- *If you were chosen to help [name of person with dementia] to make decisions, what sort of things would you need to help you?*

Supported decision-making scenarios

- *Some people think that if people choose someone to help them make decisions, the helper might end up taking over. What do you think about this? ... What could be done?*
- *Some people think that if a person has problems with their thinking or memory, that it might be safer for someone else to make decisions for the person. What do you think about this?*
- *Some people think that if people choose someone to help them make decisions, there might be disagreements or arguments. What do you think about this? ... What could be done?*
